# Supplementary figures and images for: Local resource availability drives habitat use by a threatened avian granivore in savanna woodlands
Source: PLoS One. 2024 Aug 7;19(8):e0306842. doi: 10.1371/journal.pone.0306842 (PMC11305587; doi:10.1371/journal.pone.0306842)

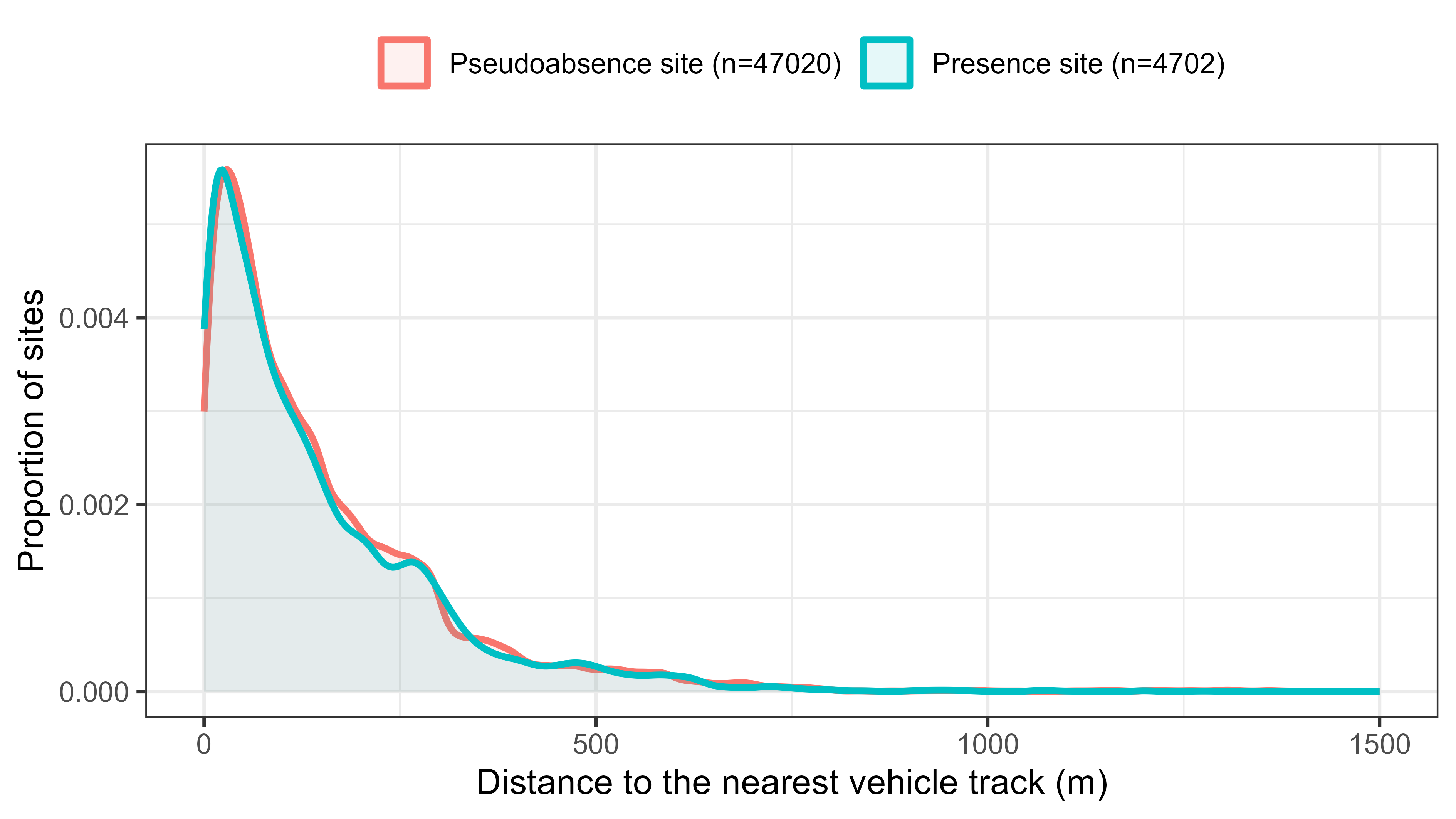

Supplement: S1 Fig — (TIF) [file pone.0306842.s001.tif]

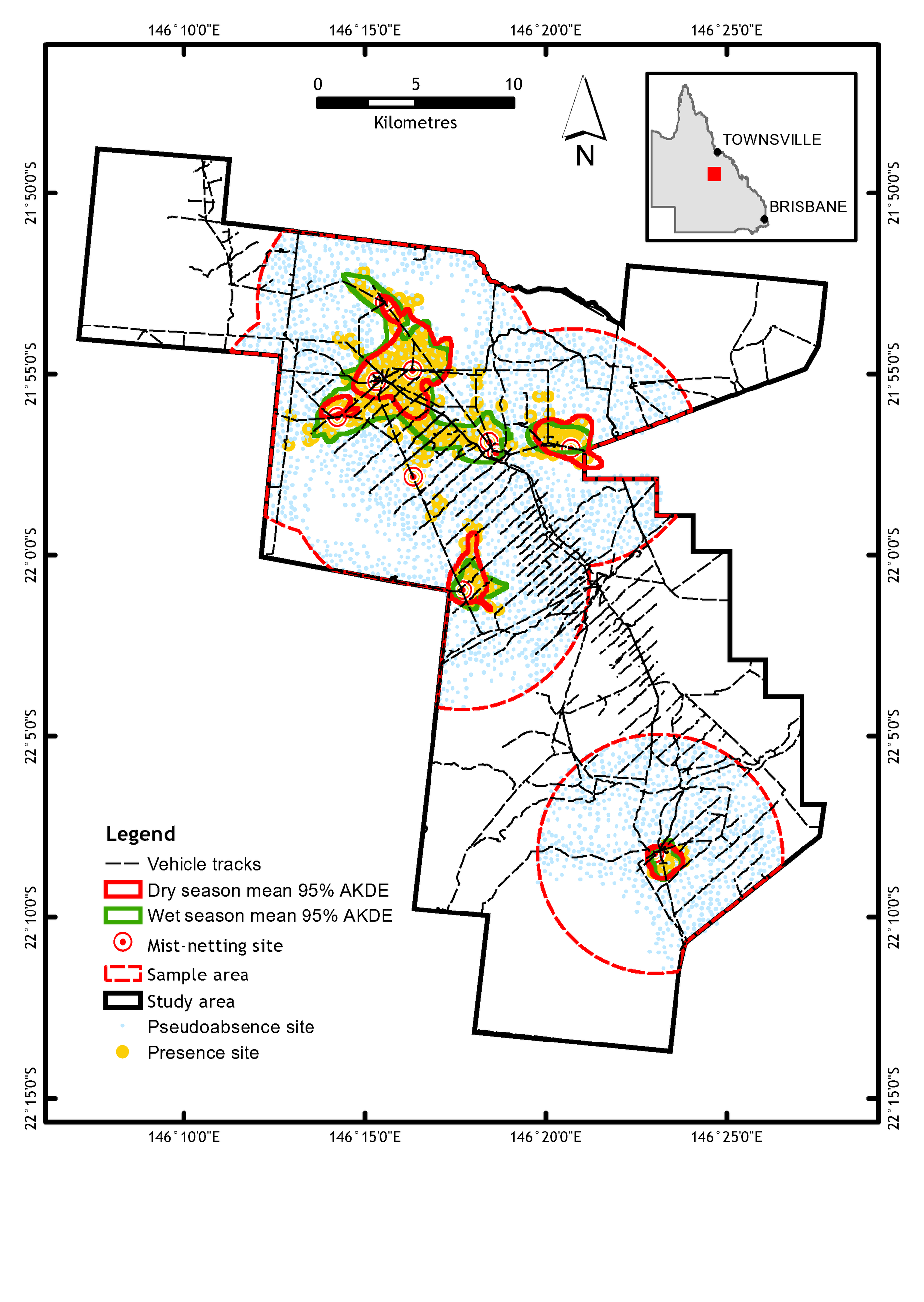

Supplement: S2 Fig — (TIF) [file pone.0306842.s002.tif]

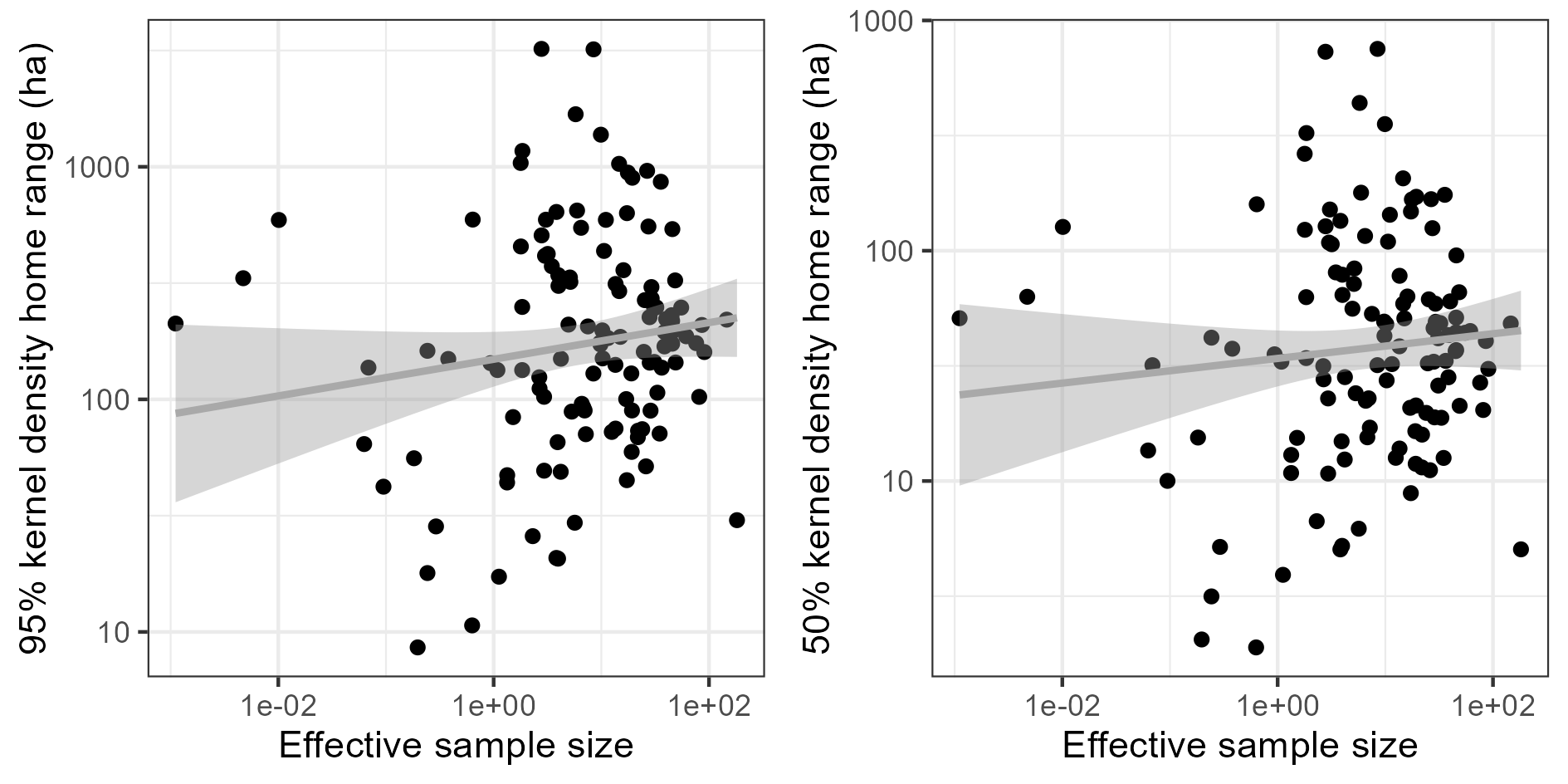

Supplement: S3 Fig — (TIF) [file pone.0306842.s003.tif]
