# Supplementary material for: Local resource availability drives habitat use by a threatened avian granivore in savanna woodlands
Source: PLoS One. 2024 Aug 7;19(8):e0306842. doi: 10.1371/journal.pone.0306842 (PMC11305587; doi:10.1371/journal.pone.0306842)
Supplement: S2 Table — (DOCX) [file pone.0306842.s005.docx]

**S2 Table.** **Vegetation communities within the study area**

| Short name | Vegetation community | Regional ecosystems* | Area (ha) |
| --- | --- | --- | --- |
| *A. shirleyi* open forest | *Acacia shirleyi* open forest on skeletal soils associated with sandstone ranges and laterite | 10.10.1 and 10.7.3 | 7,382 |
| *Corymbia* spp. woodland | *Corymbia dallachiana, C. plena* and/or *C. terminalis* woodland on sandy soils | 10.3.12 and 10.5.2 | 994 |
| *E. camaldulensis* woodland | *Eucalyptus camaldulensis* and/or *E. coolabah* open woodland to open forest associated with major water courses | 10.3.15, 10.3.13, 10.3.14, 11.3.3, 11.3.25 and 11.3.27 | 1,296 |
| *A. cambagei* woodland | *Acacia cambagei* woodland on heavy clay soils | 10.3.4, 10.4.5, 11.3.5 and 11.4.6 | 541 |
| *E. brownii* woodland | *Eucalyptus brownii* woodland on sandy plains | 10.3.6, 10.5.5x2 and 11.3.10 | 4,152 |
| *A. harpophylla* woodland | *Acacia harpophylla* woodland on heavy clay soils | 10.3.3, 10.4.3 and 11.4.9 | 2139 |
| *E. similis* woodland | *Eucalyptus* *similis* and/or *Corymbia brachycarpa* woodland on sandy plains | 10.5.1 | 16,450 |
| *E. melanophloia* woodland | *Eucalyptus melanophloia* woodland on sandy plains | 10.5.5a | 29,388 |
| *E. persistens* woodland | *Eucalyptus persistens* and *E. thozetiana* woodland on lateritic soils | 10.7.4 and 10.7.5 | 1,098 |
| *M. tamariscina* woodland | *Melaleuca tamariscina* open shrubland to low woodland on skeletal lateritic soils | 10.7.7 | 107 |
| Non-remnant | Non-remnant areas that have been cleared of native vegetation. These areas include areas converted to improved pasture dominated by exotic grasses, particularly buffel grass (*Cenchrus ciliaris*) and areas occupied by mining infrastructure and roads. | N/a | 11,945 |

* Regional ecosystems form the basis of vegetation mapping in Queensland, Australia (Neldner *et al.*, 2022), and were ground-truthed throughout the study area. Detailed regional ecosystem descriptions are available from: <https://apps.des.qld.gov.au/regional-ecosystems/>.
