# Supplementary material for: Local resource availability drives habitat use by a threatened avian granivore in savanna woodlands
Source: PLoS One. 2024 Aug 7;19(8):e0306842. doi: 10.1371/journal.pone.0306842 (PMC11305587; doi:10.1371/journal.pone.0306842)
Supplement: S3 Table — (DOCX) [file pone.0306842.s006.docx]

**S3 Table. Estimated regression parameters, standard errors, z-values and p values for a generalised linear mixed effects model relating home range size to season.**

| Predictor | Estimate | Std. error | z-value | p |
| --- | --- | --- | --- | --- |
| (Intercept) | 5.55 | 0.233 | 23.8 | < .0001 |
| Wet Season | 0.27 | 0.223 | 1.21 | 0.225 |
| Observations | 121 |  |  |  |
| R2 conditional/R2 marginal | 0.201/0.0152 |  |  |  |
