# Supplementary material for: Local resource availability drives habitat use by a threatened avian granivore in savanna woodlands
Source: PLoS One. 2024 Aug 7;19(8):e0306842. doi: 10.1371/journal.pone.0306842 (PMC11305587; doi:10.1371/journal.pone.0306842)
Supplement: S4 Table — (DOCX) [file pone.0306842.s007.docx]

**S4 Table. Estimated regression parameters, standard errors, t-values and P-values for the linear model assessing the relationship among activity, season and time of day.**

| Predictor | Estimate | Std. error | t-value | p |
| --- | --- | --- | --- | --- |
| (Intercept) | 0.00771 | 0.0353 | 0.218 | 0.83 |
| Activity (foraging) | 0.656 | 0.05 | 13.1 | < .001 |
| Activity (nesting) | 0.00337 | 0.05 | 0.0674 | 0.947 |
| Activity (perching/preening/flying) | 0.31 | 0.05 | 6.2 | < .001 |
| Activity (drinking) x Season (wet) | -0.0154 | 0.0288 | -0.534 | 0.6 |
| Activity (foraging) x Season (wet) | -0.0135 | 0.0288 | -0.469 | 0.645 |
| Activity (nesting) x Season (wet) | 0.0652 | 0.0288 | 2.26 | 0.038 |
| Activity (perching/preening/flying) x Season (wet) | -0.0363 | 0.0288 | -1.26 | 0.226 |
| Activity (perching/preening/flying) x Time of day (late morning) | 0.125 | 0.0456 | 2.74 | 0.0147 |
| Activity (perching/preening/flying) x Time of day (midday) | 0.131 | 0.0456 | 2.88 | 0.0109 |
| Activity (perching/preening/flying) x Time of day (early afternoon) | 0.243 | 0.0456 | 5.32 | < .001 |
| Activity (perching/preening/flying) x Time of day (late afternoon) | 0.0587 | 0.0456 | 1.29 | 0.217 |
| Activity (drinking) x Time of day (late morning) | 0.0375 | 0.0456 | 0.822 | 0.423 |
| Activity (drinking) x Time of day (midday) | 0.0725 | 0.0456 | 1.59 | 0.131 |
| Activity (drinking) x Time of day (early afternoon) | 0.0359 | 0.0456 | 0.786 | 0.443 |
| Activity (drinking) x Time of day (late afternoon) | 0.0455 | 0.0456 | 0.997 | 0.334 |
| Activity (foraging) x Time of day (late morning) | -0.187 | 0.0456 | -4.11 | < .001 |
| Activity (foraging) x Time of day (midday) | -0.182 | 0.0456 | -3.99 | 0.00105 |
| Activity (foraging) x Time of day (early afternoon) | -0.264 | 0.0456 | -5.8 | < .001 |
| Activity (foraging) x Time of day (late afternoon) | -0.0711 | 0.0456 | -1.56 | 0.139 |
| Activity (nesting) x Time of day (late morning) | 0.0251 | 0.0456 | 0.551 | 0.589 |
| Activity (nesting) x Time of day (midday) | -0.0218 | 0.0456 | -0.477 | 0.64 |
| Activity (nesting) x Time of day (early afternoon) | -0.0141 | 0.0456 | -0.309 | 0.761 |
| Activity (nesting) x Time of day (late afternoon) | -0.0331 | 0.0456 | -0.725 | 0.479 |
| Observations | 40 |  |  |  |
| R2 adjusted | 0.962 |  |  |  |
