# Supplementary material for: Local resource availability drives habitat use by a threatened avian granivore in savanna woodlands
Source: PLoS One. 2024 Aug 7;19(8):e0306842. doi: 10.1371/journal.pone.0306842 (PMC11305587; doi:10.1371/journal.pone.0306842)
Supplement: Attachment — Submitted filename: Response to Reviewers.docx [file pone.0306842.s008.docx]

**Response to peer review comments**

**Title:** Local resource availability drives habitat use by a threatened avian granivore in savanna woodlands

**Manuscript number:** PONE-D-24-14262

**Overview**

We thank the editor and reviewers for their constructive comments. We have addressed the comments in full, as detailed below.

**Journal Requirements**

1. **Please ensure that your manuscript meets PLOS ONE's style requirements, including those for file naming. The PLOS ONE style templates can be found at https://journals.plos.org/plosone/s/file?id=wjVg/PLOSOne_formatting_saple_main_body.pdf and https://journals.plos.org/plosone/s/file?id=ba62/PLOSOne_formatting_sample_title_authors_affiliations.pdf**

We have updated the manuscript in accordance with PLOS ONE’s style requirements, including requirements for:

- Main body formatting
- Tile, authors and affiliations formatting
- File naming conventions
- References

2. **Please note that PLOS ONE has specific guidelines on code sharing for submissions in which author-generated code underpins the findings in the manuscript. In these cases, all author-generated code must be made available without restrictions upon publication of the work. Please review our guidelines at https://journals.plos.org/plosone/s/materials-and-software-sharing#loc-sharing-code and ensure that your code is shared in a way that follows best practice and facilitates reproducibility and reuse.**

We have created a data repository in Figshare, a widely used, open access repository. The draft dataset can be accessed through this link: <https://figshare.com/s/1a0088831fc24f5d0098>

The data repository will be finalised and published following peer review comments. We will publish the data repository with the open access licence CC BY 4.0, which allows others to share, copy and modify the data provided appropriate credit is given to the authors.

The data repository contains the code and data to replicate our results. Specifically, the data repository contains:

- The code base used for statistical analyses and figure preparation (excluding figures prepared in ArcGIS (Fig 1 and S4 Fig)
- Data to support the results.

Please note that we have excluded locational information for the southern black-throated finch records. The black-throated finch is on the Queensland Government’s list of confidential species (<https://www.data.qld.gov.au/dataset/queensland-confidential-species/resource/934c92b2-3b80-4b7a-8db2-187f912de605>), whereby public-facing records are hidden for this species. As such, we have made some modifications to the R code in order to make reproducing our results possible without providing these sensitive data.

We have updated the ‘Data availability statement’ in the PLOS submission form to provide these details.

3. **Thank you for stating the following financial disclosure: "Funding provided by Bravus Mining and Resources". Please state what role the funders took in the study. If the funders had no role, please state: ""The funders had no role in study design, data collection and analysis, decision to publish, or preparation of the manuscript."" If this statement is not correct you must amend it as needed. Please include this amended Role of Funder statement in your cover letter; we will change the online submission form on your behalf.**

Thank you, we have included a revised funding statement within the cover letter. The revised funding statement now states:

“*Bravus Mining and Resources provided funding, contributed to study design and supported data collection. The funder had no role in data analysis, decision to publish or preparation of the manuscript.*”

4. **Thank you for stating the following in the Acknowledgments Section of your manuscript: "We gratefully thank Bravus Mining and Resources for their funding and support. Many staff members of E2M Pty Ltd provided administrative, logistical and field work support for the project, and we are indebted to their efforts. We also thank Juliana McCosker for long-term support of the project and of SBTF conservation broadly".**

**We note that you have provided funding information that is not currently declared in your Funding Statement. However, funding information should not appear in the Acknowledgments section or other areas of your manuscript. We will only publish funding information present in the Funding Statement section of the online submission form.**

**Please remove any funding-related text from the manuscript and let us know how you would like to update your Funding Statement. Currently, your Funding Statement reads as follows:**

**"Funding provided by Bravus Mining and Resources"**

**Please include your amended statements within your cover letter; we will change the online submission form on your behalf.**

Thank you, we have updated the Acknowledgments section within the manuscript accordingly. The Acknowledgments section now states:

“*We gratefully thank Joshua Moore, Lyndall Marshall, Andy Page, Melinda Bergmann, Samuel Wilson, Ed Meyer, Dean Jones, Chays Ogston, Jessica Hogg and Breanna Williams for their administrative, technical and field work support for the project, and we are indebted to their efforts. We also thank Juliana McCosker for long-term support of the project and of SBTF conservation broadly.*”

5. **In the online submission form, you indicated that your data is available only on request from a third party. Please note that your Data Availability Statement is currently missing contact details for the third party, such as an email address or a link to where data requests can be made. Please update your statement with the missing information.**

We have updated the Data Availability Statement in light of additional data and code being shared via an open access repository. The amended statement is copied below:

“*Data and code supporting this paper are shared through an open access repository:* [*https://doi.org/10.6084/m9.figshare.26043391*](https://doi.org/10.6084/m9.figshare.26043391)*. Sensitive locational information for the SBTF has been excluded to comply with confidentiality requirements; for further access, contact the corresponding author*”.

Please note that this is a draft dataset that will be published with a DOI following peer review comments. The draft dataset can be accessed through this link: <https://figshare.com/s/1a0088831fc24f5d0098>

6. **Please review your reference list to ensure that it is complete and correct. If you have cited papers that have been retracted, please include the rationale for doing so in the manuscript text, or remove these references and replace them with relevant current references. Any changes to the reference list should be mentioned in the rebuttal letter that accompanies your revised manuscript. If you need to cite a retracted article, indicate the article’s retracted status in the References list and also include a citation and full reference for the retraction notice.**

We have reviewed the reference listed and have made minor changes to be consistent with PLOS ONE’s reference formatting. No retracted articles were found within our review of the reference list.

Please note that we have referenced a small number of Australian and Queensland government reports and databases, which do not have an associated DOI and may cause issues in automatic reference linking. These reports are the best source of information for some key information in this study (primarily threatened species’ status and climate/habitat information).

**Additional Editor Comments**

1. **Both reviewers mentioned the lack of availability of your data in the initial section of questions. Maybe, you can add some basic data as Supplementary Material. Please check Instructions about this again to verify what is better for you, but informative and important for authors.**

We have added a data repository in Figshare, a widely used, open access repository. The draft dataset can be accessed through this link: <https://figshare.com/s/1a0088831fc24f5d0098>

The data repository will be finalised and published following peer review comments. We will publish the data repository with the open access licence CC BY 4.0, which allows others to share, copy and modify the data provided appropriate credit is given to the authors.

The data repository contains the code and data to replicate our results. Specifically, the data repository contains:

- The code base used for statistical analyses and figure preparation (excluding figures prepared in ArcGIS (Fig 1 and S4 Fig).
- Data to support the results.
- Figures and tables listed in the supporting information section of the manuscript.

Please note that we have excluded locational information for the southern black-throated finch records. The black-throated finch is on the Queensland Government’s list of confidential species (<https://www.data.qld.gov.au/dataset/queensland-confidential-species/resource/934c92b2-3b80-4b7a-8db2-187f912de605>), whereby public-facing records are hidden for this species. As such, we have made some modifications to the R code in order to make reproducing our results possible without providing these sensitive data.

We have updated the Data availability statement’ in the PLOS submission form to provide these details.

Additional note: Our code had randomly selected pseudoabsence sites to use in the habitat selection model (Fig 3). We had to re-run the random selection process and resulting model, which resulted in very minor differences in the model output coefficients presented in the Landscape Scale Habitat Use section of the results. The differences in model outputs were all much less than 1% and do not affect the conclusions reached in any way. All changes have been included in the ‘Revised Manuscript with Track Changes’.

2. **Title: I´m a bit worried with the use of the term "selection". Have you examined it ? Ususally, selection involves the measurement of habitat use and availability. Please talk with the co-authors again about this and follow what is better for you.**

Thank you, we had incorrectly conflated habitat selection with habitat use, which you and reviewer 2 both identified. We have updated the text to refer to ‘habitat use’ throughout the manuscript, including within the title.

3. **Formatting:**
   a. **You are using round brackets to cite references. It is wrong. You have to use square brackets: So, use, for example, [2] instead of (2). Here and along all parts of the text.**
   b. **Also, when you cite two references, you have to add a space after the comma. Then you will have [2, 3].**
   c. **Also, you are using hyphen to separate two consecutive numbers of references, but you have to use a long dash. Please see recent papers for comparison.**

We have updated the manuscript to make the identified formatting updates in full. We have also reviewed all usage of En dashes, Em dashes and hyphens to make sure they are consistent and within PLOS ONE’s formatting standards.

4. **Line 85-94. Paragraph of the objective. Please note that here you use "habitat use", not selection. Have to check and decide.**

Thank you, we have updated the text throughout to refer to ‘habitat use’, as recommended.

5. **Material and Methods:**
   a. **Line 98. You have to use "(Fig 1)". Abbreviated and without dot. Please check this for all figures along the paragraphs.**

Updated for this line and throughout the document.

b. **Line 106. Add 1-2 refs at the end.**

We have added a reference to Fig 1, which now shows the Carmichael River (discussed in this sentence) and also recommended by Reviewer 1.

c. **Line 109. I think the correct is "(Fig 1, S2 Table).**

Amended accordingly.

d. **Line 177-119. Use "Fig 1." in bold in captions. It is better if you complete the first sentence by citing the full species name, savannas, the Australian region.**

Caption amended to:

(Lines 119–123) **“*Fig 1. Study area for Southern Black-throated Finch research in savanna vegetation within the Desert Uplands region of Queensland, Australia.*** *The map depicts the locations of automated radio towers, mist-netting sites, vegetation communities, major roads, major watercourses and mining infrastructure. Abbreviations include A = Acacia, E = Eucalyptus, M = Melaleuca, O.F. = open forest and W = woodland.*”

In addition, we have reviewed captions throughout the manuscript and ensured they follow PLOS ONE’s formatting requirements.

e. **Line 137. Use just "(S1 Table)". Please follow this for all tables and figures as supplementary material.**

Thanks, we have reviewed and updated all references to supporting information accordingly.

f. **Lines 173-174. "towers" appears four times in two lines. Could you make some changes to avoid these repetitions ?**

Amended accordingly to:

(Lines 178–180) “*The setup included four arrays of four towers each, and an additional array of 11 towers that comprised seven units the centre surrounded by four units in peripheral areas (Fig 1).*”

6. **Results:**
   a. **Line 302. Use "Fig 2." in bold in captions. Add the Australian region at the end of the first sentence.**

Caption amended to:

(Lines 312–315) **“*Fig 2. Histogram showing the distribution of radio tracked Southern Black-throated Finch in the Desert Uplands region of Queensland, Australia****. Resightings are relative to the distance from their point of capture and include resightings from automated radio telemetry (ART, red) and manual radio tracking (blue).*”

b. **Line 306. Use "(Fig 3).". Please check this for all figures.**

Amended and updated for all figure references.

c. **Line 320. Inform the Australian region. Also, you have to explain in the caption what are these scientific names, O.F. and W.**
d. **Explain the red and blue colors. Delete the SBTF abbreviation after the full name.**

Thanks, we have updated the figure caption to address both 6c and 6d. The caption now reads:

(Lines 332–337) **“*****Fig 3. Odds ratios of the predictor variables of a logistic regression resource use model in the Desert Uplands region of Queensland, Australia.*** *Odds ratios above 1 represent a use preference by Southern Black-throated Finch (blue), whereas odds ratios lower than 1 represent avoidance (red). The error bars show 95% confidence intervals. ***p < 0.001, **p < 0.01, *p < 0.05. Abbreviations include A = Acacia, E = Eucalyptus, M = Melaleuca, O.F. = open forest and W = woodland.”*

e. **Line 375. Figure 5. Some people will not distinguish the red and green colors. Consider changing some colors here and in other figures.**

Thank you for this recommendation. We have amended Figures 4 and 5 to be accessible for readers with colour vision deficiencies. The colours we have used follow R’s version 4.0.0 colour palette, which have been designed to provide distinguishable colours robust to colour vision deficiencies (<https://journal.r-project.org/articles/RJ-2023-071/>). In addition, we reviewed all other figures in this manuscript and supporting information were reviewed.

7. **Discussion: It appears to be well divided in sections and very well written.**

Thank you

8. **References: They are partly formatted. I think you have to do the following changes:**
   a. **After the year, you must have a semi-colon and a space "2021; 15:"**
   b. **You must have a space prior to the pages, and they have to be separated by a long dash, not hyphen.**
   c. **It is better if you add the DOI for all the refs that have it.**
   d. **Please check the Instructions again.**

We have updated the references to make these changes in full. We have also reviewed PLOS ONE’s referencing instructions and made various minor updates to the references for consistency with the instructions.

**Review #1 Comments**

1. **Line 22 - state the number of towers used**

Updated, thank you. New text reads:

(Line 23) *“…an array of 27 automated radio towers…”*

2. **Line 87-88 - perhaps move this information to the methodology section**

We have updated the manuscript to move that sentence to the start of the manual radio tracking section in the methodology (lines 154–156), as recommended. We also made minor edits to the paragraph which the lines were extracted from (lines 86–92) to improve clarity and coherence.

3. **Line 101-102 - if possible, add mining infrastructure, major roads, Carmichael River, and other features that may influence habitat selection to the map**

We have updated Fig 1 to show all of these features.

Please note that in addition to the layers in Fig 1, the property track network is mapped in S3 Fig. We excluded the property track network from Fig 1 as it made the figure cluttered.

4. **Line 115 - if possible add average distance of mining infrastructure to ART towers or acknowledge distance of banding stations/towers to mining area.**

We have added this information to lines 182–183, under the Automated Radio Telemetry section of the methods.

**“***The ART towers were on average 10.3 km (range: 5.03–14.9 km) from the nearest mining infrastructure.*”

In addition, the update to Fig 1 now shows the location of mining infrastructure relevant to our sampling areas.

5. **Line 116 - clarify if non-remnant is all mining area or if it's something else**

We have updated Fig 1 (a map of the study area) to show the extent of mining infrastructure. In addition, lines 115–117 have been updated to provide more detail on the composition of non-remnant areas:

*“In addition, non-remnant areas, which have been cleared of native vegetation [27], cover 21% of the study area. Of the non-remnant areas, 16% are used for cattle grazing pastures and 5% are occupied by mining infrastructure and roads (Fig 1).”*

6. **Line 143 - explain ART acronym**

Thanks, we have edited this to ‘automated radio telemetry’ (lines 145–146) and introduce the acronym later in the methods (line 173) when it is used more frequently.

7. **Line 132 - was the number of BTF banded the same as the number of BTF tagged?**

We have an existing sentence in lines 150–152 stating that “*All SBTF captured were also fitted with a uniquely coded Australian Bird and Bat Banding Scheme supplied alloy band.*”

The broader research program (outside of this manuscript) includes a colour-banding program that is investigating the long-term movements of the species; particularly looking at their sedentary to nomadic behaviours. In answer to your question: the number of BTF banded was actually more than the number tagged but that is because of additional BTF tagged for research questions not included within this manuscript. We have therefore opted to only provide information for those birds used for the data in this manuscript. No changes have therefore been made but please revert if you would like to see more of a discussion included about this.

Please note that we have maintained reporting obligations for all birds captured through mandatory reporting requirements under our Research Permit (reported to the Queensland Government), Australian Bird and Bat Banding Scheme Permit (reported to the Australian Government) and Animal Ethics Permit (reported to the Queensland Government).

8. **Line 151 - add details on how many BTF were manually tracked, how many BTF were tracked for 5hs, etc.**

We have included information on the number of BTF manually tracked within the results section, specifically lines 298–301, which state that:

***“****For each of the 142 radio tracked SBTF, we recorded an average of 1890 ± 4180 ART locations (range = 0–45600) and 11.9 ± 11.9 manual radio tracking locations (range = 1–57). All SBTF were detected through manual tracking at least once, while eight SBTF were not detected on the ART receivers post-release.”*

We have added additional information to lines 301–302, to provide more information on the range of manual tracking periods:

*“The average number of manual radio tracking detections per day that the individual was detected was 3.3 (range = 1–30)”.*

It is difficult to state exactly how many birds were tracking as single detections vs 5hr detections, etc.. as it was highly variable and largely depended on how long the human observer could stay in contact with the flock for. We also noticed an error in the manuscript on line 163, which said that *‘tracking bouts ranged from single sightings to approximately 5 hours of tracking per bird’*. This should have been 8 hours and we have updated it accordingly. My apologies for the oversight.

9. **Line 171 - "throughtout" gives the idea that towers were homogeneously distributed and not grouped. Rewrite to clarify and add information about what vegetation community the towers were placed in.**

Agreed, we have amended the text accordingly, to:

(Lines 177–178) *“We installed the ART towers within remnant vegetation in areas of known foraging, drinking and nesting habitats for SBTF.”*

10. **Line 468 - Rechetelo et al. did a rough analysis of the activity patterns on the ground, maybe you can confront the information.**

Thank you for the recommendation. We have elaborated on this point and extended this consideration to a foraging ecology study undertaken by Mitchell in 1992. We have included this within the discussion section in lines 478–483, which now read:

“A bimodal peak in forging activity has been identified for other Australian estrildid finches [52]. However, SBTF specific studies have reported varying results. Using 659 activity observations of SBTF, Mitchell [56] did not find a daily pattern to foraging activity, while in contrast, a coarse analysis of 620 activity observations by Rechetelo et al. [6] suggested a pattern of bimodal foraging similar to our study.”

**Review #2 Comments**

1. **Please revise the manuscript to increase use of active voice.**

We have updated a range of text throughout the document to increase the use of active voice. Specific updates include:

- Line 90: “*More broadly, our research aims to…*”
- Line 95: *“We conducted this study…”*
- Line 163: *“We collected the location of tracked SBTF after**…”*
- Line 168: *“We recorded the GPS locations of tracked SBTF…”*
- Line 177: *“We installed the ART towers within…”*
- Line 184: *“Automated radio telemetry data require…”*
- Line 191: *“After filtering, we arranged the detections by…”*
- Line 216: *“We generated pseudoabsence sites within…”*
- Line 229: *“We mapped the distributions of…”*
- Line 243: *“We converted model estimates to…”*
- Line 317: *“At the landscape scale, we observed that SBTF…”*
- Line 330: *“We resighted radio tracked SBTF…”*
- Line 326: *“Southern black-throated finch habitat use was strongly related to…”*
- Line 339: *“We were able to fit utilisation distributions (UDs) in ctmm for…”*
- Line 354: *“We recorded 1054 SBTF activity observations…”*

2. **I suggest a careful revision of word choice when describing habitat use vs selection. To my understanding of your sampling and analytical approach, your paper evaluates habitat use of black-throated finches and not selection as you are not analytically determine use versus availability of habitat types (i.e., land-cover types). As a result, you need to replace “selection” with “use”. If my understanding is incorrect, please pardon the oversight, then you need to add text describing in detail that your study evaluates habitat selection and not use.**

Thank you, we had incorrectly conflated habitat selection with habitat use, which both reviewer 2 and the editor identified. We have updated the text to refer to ‘habitat use’ throughout the manuscript.

3. **Please provide a more detailed description of ecosystem types for readers who have not had the opportunity to experience them.**

We have substantially expanded on the description of ecosystem types in lines 103 to 117, as follows:
*“Remnant vegetation covers approximately 79% of the study area and includes 10 vegetation communities that we assigned based on similarities in dominant canopy species and soil attributes (Fig 1, S2 Table). These vegetation communities are based on the Queensland Government’s regional ecosystem framework [27], which was extensively ground-truthed throughout the study area. Broadly, the study area’s vegetation comprises semi-arid savanna woodlands that are dominated by eucalypt and Acacia species [17]. Eucalypt woodlands account for 71% of the remnant vegetation, with the most common communities being those dominated by silver-leaved ironbark (Eucalyptus melanophloia) (39%) and yellow jacket (E. similis) (22%) on deep red earths (Fig 1, S2 Table). Other remnant habitats include lancewood (Acacia shirleyi) on skeletal Tertiary plateaus, E. camaldulensis and E. brownii woodlands on alluvial plains associated with watercourses and drainage features, brigalow (A. harpophylla) and gidgee (A. cambagei) open forests on clay plains, and small areas of Melaleuca dominated shrublands (Fig 1, S2 Table). In addition, non-remnant areas, which have been cleared of native vegetation [27], cover 21% of the study area. Of the non-remnant areas, 16% are used for cattle grazing pastures and 5% are occupied by mining infrastructure and roads (Fig 1).”*

In addition, more detailed descriptions of the vegetation communities within the Study Area are provided in S2 Table. We have added a link to an open access data source to provide even more extensive information on the vegetation communities that we discuss.

4. **When did you commence data collection after capture? Did you use the commonly applied 24-hr waiting period?**

Yes, we did apply a 24 hour waiting period. We have added a statement to lines 156–157 to address this:

*“**We excluded tracking data obtained within 24 hours of transmitter attachment from all statistical analyses to allow a post-release acclimation period.”*

5. **Please provide links to R code and raw data for readers to replicate analyses. If locations of black-throated finches cannot be made public due conservation concerns, please state so.**

We have added a data repository in Figshare, a widely used, open access repository. The draft dataset can be accessed through this link: <https://figshare.com/s/1a0088831fc24f5d0098>

The data repository contains the code and data to replicate our results. However, please note that we have excluded locational information for the southern black-throated finch records. The black-throated finch is on the Queensland Government’s list of confidential species (<https://www.data.qld.gov.au/dataset/queensland-confidential-species/resource/934c92b2-3b80-4b7a-8db2-187f912de605>), whereby public-facing records are hidden for this species. As such, we have made some modifications to the R code in order to make reproducing our results possible without providing these sensitive data.

The data repository will be finalised and published following peer review comments. We will publish the data repository with the open access licence CC BY 4.0, which allows others to share, copy and modify the data provided appropriate credit is given to the authors.

6. **Line 26: Provide range of tracking periods. The SD is not informative other than to demonstrate that there is a lot of variation in tracking periods.**

Thank you, we have made the update as recommended.

(Line 27) *“…radio tracking period (21.8, range = 0.83–120 days)”.*

7. **Line 40: Replace “selection choice” with “use”.**

Thank you, we have made the update as recommended.

8. **Line 77: Refer Fig. 1 here as readers unfamiliar with the geography of Australia may not know the location of the Townsville Coastal Plains.**

Thank you, we have made the update as recommended.

9. **Line 147: SD format differs from above.**

Thank you, we have made the update as recommended.

(Line 149–150) *“…which was 21.8 ± 22.5 days (*$\bar{x}$ *± 1 SD , range = 0.83–120 days)…”*

10. **Line 206: Describe the process of how you selected two presence sites per day? Did you use a stratified approach where observations were separated by a certain length of time or restricted to morning vs evening?**

The subsampling process was random, with no stratification applied. A minimum sampling interval of 15 minutes was used for both datasets to make them equivalent.

We have updated the text in lines 213–216 to state that:

*‘Presence sites were randomly subsampled to a maximum of two sites per day from each of the manual radio tracking and ART locations to minimise bias from auto-correlation of radio tracking data [6, 37]. The minimum time allowed between ART subsamples was 15 minutes, which matched the minimum sampling frequency of manual radio tracking.’*

11. **Line 209: Explain your rationale for increasing pseudo-absence points by a factor of 10 compared number of presence sites.**

We have added a reference to the study that provides recommendations on the optimal number of pseudo-absence sites to model within line 219 of the manuscript.

The sentence now reads (lines 216–219): *“We generated pseudoabsence sites within a subset of the study area that we defined as the ‘sample area’ (Fig 1) at a 10x multiple of presence sites using ArcGIS Pro (ESRI), which followed recommendations by Barbet-Massin et al. [39].”*

We note that a 10x multiplier seems to be common practice, evidenced by its use as the default pseudoabsence multiplier in the animal movement tools R package (<https://doi.org/10.1002/ece3.4823>).

12. **Line 246: Provide criteria when subsampling ART locations.**

Thanks, similar to item 10 above, we have updated the text in lines 253–255 to state that:

*“…we subsampled the ART location estimates to two random locations per day, with a minimum of 15 minutes between subsamples, which approximated the sampling frequency of the manual radio tracking dataset.”*

13. **Line 286: Provide general statement here stating that data are reported as** **mean +/- SD (range). This will decrease repeated verbiage in the Result section.**

Thanks, this is a much cleaner approach. We have added a sentence, as recommended, which states that (lines 294–295) *‘Unless otherwise specified, data in the results section below are reported as mean ± SD (range)’.*

We have amended text in the results section accordingly.

14. **Line 386: Define “non-remnant” in Method section.**

We have added the definition of non-remnant to lines 115–117, as follows:

*“**In addition, non-remnant areas, which have been cleared of native vegetation [27], cover 21% of the study area. Of the non-remnant areas, 16% are used for cattle grazing pastures and 5% are occupied by mining infrastructure and roads (Fig 1).”*

15. **Figure 3: See general comment 3. Also color legend to text: … used (blue)**

Updated as per general comment 3. We have also added text to describe colours within the figure, as recommended.

Our sincere thanks for your constructive comments and we look forward to the next steps.

Kind regards

John van Osta
